# Case series of 589 tooth extractions in patients under bisphosphonates therapy. Proposal of a clinical protocol supported by Nd: YAG low-level laser therapy

**DOI:** 10.4317/medoral.18812

**Published:** 2013-03-25

**Authors:** Paolo Vescovi, Marco Meleti, Elisabetta Merigo, Maddalena Manfredi, Carlo Fornaini, Rebecca Guidotti, Samir Nammour

**Affiliations:** 1 DDS, MSc, Associate Professor, Director of the European Master Degree on Oral Laser Applications (EMDOLA). Department of Biomedical, Biotechnological and Translational Sciences - S.Bi.Bi.T; University of Parma - Italy; 2 DDS, PhD, Consultant Professor, Dental Faculty, Department of Biomedical, Biotechnological and Translational Sciences - S.Bi.Bi.T; University of Parma - Italy; 3 DDS, MSc, Consultant Professor, Dental Faculty, Department of Biomedical, Biotechnological and Translational Sciences - S.Bi.Bi.T; University of Parma - Italy; 4 DDS, PhD, Assistant Professor, Dental faculty, Department of Biomedical, Biotechnological and Translational Sciences - S.Bi.Bi.T; University of Parma - Italy; 5 MD, DDS, MSc, Consultant Professor, Dental Faculty, Department of Biomedical, Biotechnological and Translational Sciences - S.Bi.Bi.T; University of Parma - Italy; 6 DDS, MSc, Resident, Dental Faculty, Department of Biomedical, Biotechnological and Translational Sciences - S.Bi.Bi.T; University of Parma - Italy; 7 DDS, MSc, PhD, Full Professor, European General Director of the European Master Degree on Oral Laser Applications (EMDOLA). Dental Faculty - University of Liège - Belgium

## Abstract

Objective: Trauma during dental surgery is a predisposing factor for bisphosphonates (BP)-related osteonecrosis of the jaws (BRONJ). However, about 40% of cases of BRONJ are not related to dental invasive procedures, being probably associated to endodontic or periodontal infections. Extraction of non-treatable teeth is considered a reliable choice, to improve symptoms and to reduce the risk of BRONJ. Here we report our experience of tooth extractions in patients under oral or intravenous BP therapy. Study Design: Two-hundred and seventeen patients (38 males, 179 females; mean age 68.72 ± 11.26 years, range 30 to 83 years) under BP therapy received 589 tooth extractions at the Unit of Oral Medicine, Pathology and Laser-assisted Surgery of the University of Parma, Italy, between June 2006 and December 2010. Ninety five patients were under BP therapy for oncological disease (multiple myeloma (MM): 23; bone metastases (BM): 72) and 122 patients for non oncological diseases: 119 osteoporosis (OP), 2 rheumatoid arthritis (RA) and 1 Paget’s disease (PD). The mean duration of BP was of 35 months. Antibiotic treatment was administered three days before and 2 weeks after tooth extractions. Patients were additionally treated with low level laser therapy (LLLT) through Nd:YAG laser (1064 nm – power 1.25 W; frequency 15 Hz; fibre diameter: 320 ?m), 5 application of 1 minute each. Patients were evaluated 3 days and once a week for 2 months after the extractions and every time they received LLLT. Mean follow-up was 15 months (ranging from 4 to 31 months). Results: In a total of 589 extractions (285 mandibular, 304 maxillary) performed, a minimal bone exposure was observed in 5 cases, treated with Er:YAG laser vaporization and than healed. Conclusions: Our experience supports the hypothesis that the association of antibiotic treatment and LLLT can be effective in preventing ONJ after tooth extractions in patients under BPT.

** Key words:**Nd:YAG laser, low level laser therapy, tooth extractions, bisphosphonates, jaws osteonecrosis.

## Introduction

The avascular osteonecrosis of the jaws (ONJ) associated with bisphosphonates (BP) therapy was firstly described in 2003 (1). Patients under oral BP have a lower prevalence of ONJ (0.01% to 0.04%) than those teated intravenously (0.8% to 12%). In particular, ONJ seems to occur most frequently in patients using BP for multiple myeloma (MM), bone metastases (BM) and osteoporosis (OP) (2).

Trauma during dental surgery is a well-recognised predisposing factor for BP-associated ONJ (BRONJ): if 60% of cases occur after oral surgery including tooth extractions, about 40% of BRONJ is not related to dental procedures (3,4). In their review, Marx et al. identified several co-morbidity factors such as presence of periodontal diseases, decays and dental abscesses. Age and prolonged BPT have also been associated with an increased risk of BRONJ (5).

Main clinical BRONJ manifestations include presence of persistent bone exposures, possibly associated with presence of fistulas, purulent discharge, pain, paraesthesia, loss of teeth and mandibular fractures. About 65% of patients presents mandibular lesions (6).

Ruggiero et al. proposed a clinical staging system for BRONJ, which recognises 3 stages: stage I - asymptomatic exposed bone and no soft tissues infection; stage II - exposed bone, pain, infection and swelling of the soft tissue; stage III - exposed bone, pain, pathological fractures and soft tissue infections (7). Presence of avascular necrotic bone, bacterial and fungal colonies as well as abundant inflammatory infiltration are typical histopathological findings (8).

BP inhibit turnover and repairing capacity of the bone after micro damages (9), reducing also the epithelial cells proliferation rate in vitro, via the reduction of farnesyl diphosphate synthetase, and exhibiting also antiangiogenetic properties by decreasing the production of vascular endothelial growth factor (VEGF) (10,11), thus determining important effects on both quality and quantity of bone vascularization, possibly altering the response to trauma and infections (12).

Several authors studied the relationship between BRONJ and dental procedures, and proposed clinical protocol for preventing the occurrence of (13,14). However, evidence-based guidelines for the management of dental extractions in patients under BP therapy (BPT) are still lacking.

Currently, low-level laser therapy (LLLT) is employed in a wide range of cutaneous, mucosal and bone disorders with bio-modulative and analgesic purposes.

We recently observed and reported the usefulness of Nd:YAG laser biostimulation associated with medical or surgical therapy in the management of BPT-associated ONJ. On the basis of the above mentioned results, we hypothesized that LLLT may also improve the post-extractive healing process in patients under BP therapy (15,16).

## Patient and Methods

Five-hundred eighty-nine tooth extractions were performed in 217 patients (38 males, 179 females; mean age 68.72 years, range 30 to 83 years) under BPT at the Unit of Oral Medicine, Oral Pathology and Laser-assisted Oral Surgery of the University of Parma, Italy, between June 2006 and December 2010.

Two-hundred seventy-one tooth extractions were performed in 95 cancer patients (23 multiple myeloma, 72 Bone metastasis). In 87 cases administered BP drug was zoledronate, in 1 case zoledronate and pamidronate, in 3 cases aledronate, in 1 case risedronate and in 3 cases aledronate and zoledronate. One hundred and twenty-two non-cancer patients underwent 318 extractions.

In 51 cases administered BP drug was aledronate, in 24 clodronate, in 17 risedronate, in 30 cases different association of BPs.

Mean duration of BPT before the extractions was 17 months for the oncological patients, 53 months for the osteoporotic patients (ranging from 1 to 92 months).

On the basis of medical conditions, the specialists suggested BPT discontinuation for 2 months before and after tooth extractions in 49 Patients.

Twenty patients out of 217 were affected by BRONJ occurring at a oral subsite not related with the area of dental extraction.

For each patient were considered adjunctive pharmacological therapies and presence of risk factors for BRONJ such as smoking habit, diabetes, vascular diseases and renal failure. Orthopantomography and, in cases of necessity, intraoral radiographies were obtained for all patients.

Tooth extractions were performed when no conventional dental treatment would have been reliable, and particularly in case of: diffuse and symptomatic periodontal and/or endodontic infections; destroying caries; complicated dental fractures. Five-hundred eighty-nine teeth were extracted under local anaesthesia, in agreement with the oncologist and/or the general practitioner of each patient. All patients were informed about the individual risk of BRONJ occurrence after oral surgical procedures, and a written consensus was obtained in each case.

One week before tooth extraction professional oral hygiene procedures were performed. Amoxicillin (2 grams per day) was administered 3 days before and for 2 weeks after tooth extractions until suture removal. Post-extractive sockets irrigations with povidone-iodine were administered. Mouthwashes with chlorhexidine 3 times per day were recommended until complete mucosal healing.

Patients received LLLT (Nd:YAG laser, 1064 nm, Fidelis Plus, Fotona®, Slovenia – power:1.25 W; frequency: 15 Hz; diameter of the fibre: 320 ?m) which was administered in non-focused mode, at 2 mm of distance from the tissues, for 1 minute (power density: 1562.5 W/cm2, total fluence 7 J/cm2), repeated 5 times. Intra-operative LLLT was delivered in the post-extractive sock-ets once a week for 6 times. Patients were evaluated after 3 days and once a week for 2 months after tooth extraction. After this period, follow-up scheme included monthly clinical evaluations as well as radiological examination every six months ( Table 1).

**Table 1:**
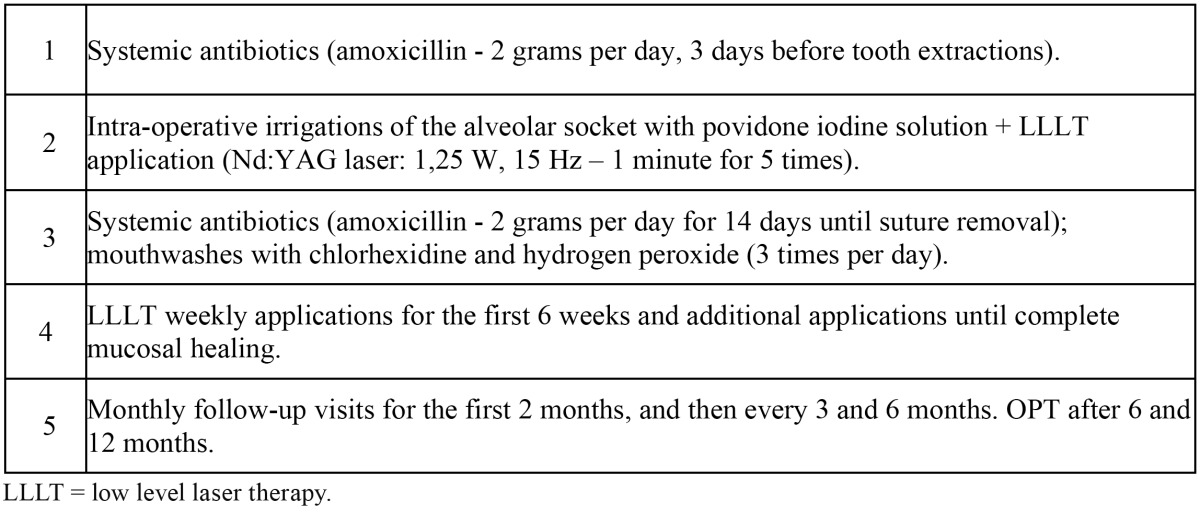
Clinical protocol used in the present study for tooth extractions in patients under BPT.

Surgical procedures were performed according to the protocol of Parma University Hospital.

## Results

Five hundred eighty-nine dental extractions (285 mandibular, 304 maxillary) ( Table 2) were performed. 150 out of 589 dental extractions were performed through the use of mucoperiosteal flaps and osteotomy. Fifteen patients have had a delayed but complete healing of the post-extractive sockets in a maximun period of 8 weeks and a minimal bone exposure was observed after 5 tooth extractions performed in cancer patients (4 male, 1 female) among a total of 589, with an incidence about 0,85% ( Table 3).

**Table 2:**
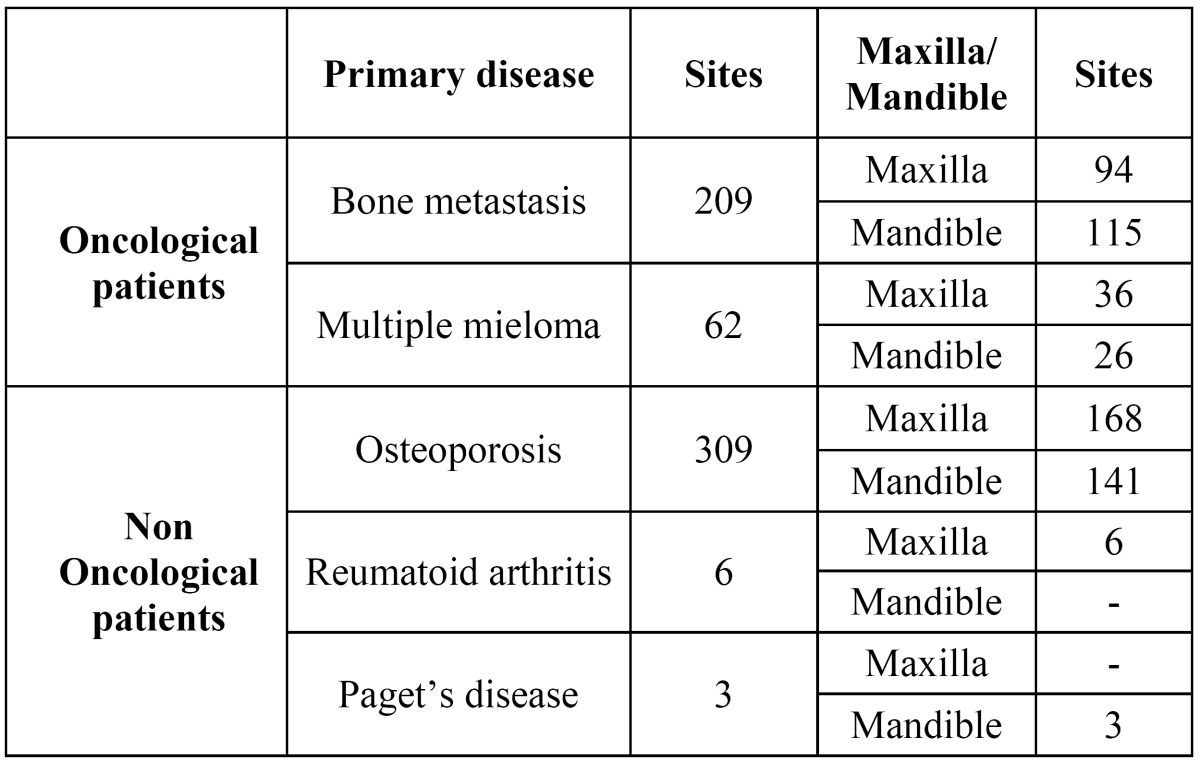
Characteristics of extraction sites: number of extraction sites, primary disease and maxillary or mandibular localization.

**Table 3:**
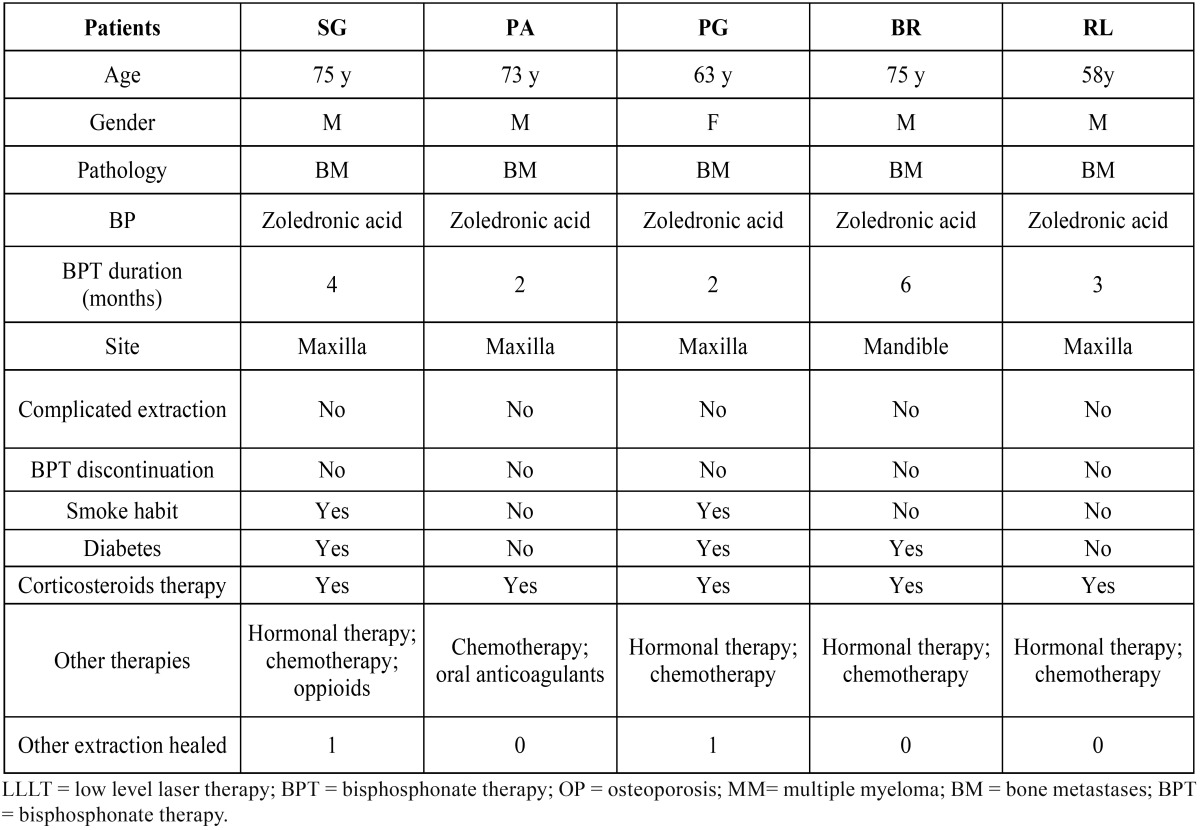
Clinical data of 5 patients with difficult healing after tooth extractions.

Such post-extractive sites were characterized by the presence of small fragments (less than 3mm in the main size) of bone, partially covered by non-inflamed mucosa. Pain and pus discharge were absent in all cases. Management of these complications was based on antiseptic, adjunctive antibiotic therapies (amoxicillin, 2 grams per day and metronidazole, 500 milligrams per day) and LLLT.

Wounds closure was obtained within 2 months through a single intervention of curettage and vaporization with Er:YAG laser (2940 nm, 250 mJ, 20 Hz, VSP, Fluence 50 J/cm2, Fidelis Plus, Fotona®, Slovenia) of the little bone residual fragments. Clinical data including risk factors and medical conditions of these patients are summarized in  Table 3.

## Discussion

Tooth extractions is a well known risk factor for BRONJ development, but an increasing of non-triggered forms has been reported in the literature (3).

In an Italian multicentric study 205 out of 567 patients (36,2%), evaluated by Vescovi et al had a non-triggered bone exposure, probably related to endodontic and/or periodontal infections (17). It can be hypothesized that the action of oral bacteria altogether with a decreased healing potential of the bone and soft tissues induced by BPT may favourite the development of BRONJ (18).

Guidelines for the dental management of patients under BPT recommend, in cases of infective odontogenic foci, to avoid dental surgery and suggest medical and conservative approaches (14,19). However, prolonged antibiotic treatments and complex dental rehabilitations may not be a reliable option for oncologic and compromised patients (20). These considerations lead to the question whether it is preferable to extract or instead to restore extremely compromised teeth taking into account the risk of BRONJ development.

Furthermore, the effective role of prophylactic antibiotic treatment has never been established.

Antibiotic treatment we used in our BPT patients was chosen on the basis of literature protocols for both type of antibiotic (amoxicillin or an alternative broad-spectrum antibiotic for subjects allergic to penicillin) and schedule (every 12 hours starting three days before the surgical procedure and for 14 days) (ie, until the second control visit and suture removal). Dentists, oral surgeons and oncologists should carefully consider risks and benefits of possible BPT discontinuation before and after dental invasive procedures (17). Cessation of BPT may expose patients to malignancy-associated hypercalcaemia, failure of control of the skeletal related events (SRE) and osteoporosis progression (21).

Our experience confirms that BPT discontinuation does not affect the long-term results of oral surgical procedures with regard to the occurrence or worsening of BRONJ (22).

In the present analysis, wound healing after tooth extractions was independent from presence or absence of BRONJ, from the anatomic site treated (maxillary or mandibular), from medical conditions and from BPT administration modality.

According to the American Academy of Oral and Maxillofacial Surgeons (AAOMS) and to the American Dental Association (ADA) guidelines, tooth extractions in patients under BPT should be minimally invasive, with limited bone and soft tissues manipulation (23). Muco-periosteal covering of post-extractive sockets are also recommended (19).

The biostimulatory effects of LLLT performed through different wavelengths on the trophism of bone and mucosa, both in vivo and in vitro, have been evaluated by several Authors, even in case of tooth extractions in BPT patients (24). Reported phenomena include faster wound healing and increased fibroblast, chondroblasts proliferation, collagen synthesis, stimulation of osteogenesis, bone cells differentiation and bone repair mechanisms, increasing of blood flow, stimulation of endothelial cells proliferation and reduction of pain (25-27); Kucerova et al. evaluated 150 patients receiving or not LLLT after lower third molar extractions, demonstrating the usefulness of laser biostimulation (28).

Lodi et al referred that none of 23 patients under BPT (without BPT discontinuation) receiving tooth extractions, developed ONJ in a follow-up period of 15 months (29). Saia reported an occurrence of BPT-related BRONJ in 5 out of 60 high risk patients (8,3%) receiving tooth extractions. In every case BPT was suspended for 1 month after surgery to improve bone healing. The authors realized biopsies in post-extractive sockets during tooth extractions to assess bony features and to rule out asymptomatic BRONJ. In most cases they found normal or minimally altered bone histology and none of these minor alterations progressed to BRONJ. In 5 patients bone biopsy specimen were positive for osteomyelitis at baseline and were associated in all cases with the occurrence of clinical or radiologic BRONJ during 12 months of follow-up (30).

## Conclusions

The results reported in the present evaluation suggest that tooth extractions should not necessarily be avoided during BPT, most of all if there are no reliable alternatives. The authors conclude that the association of antibiotic treatment and LLLT described here and the suggested prophylactic protocol, has been effective, for reducing the incidence of BRONJ after tooth extractions and limit the spread of odontogenic infections in patients already debilitated by systemic disease.
